# The effects of refractive status on the outcomes of strabismus surgery in patients with esotropia

**DOI:** 10.1186/s12886-024-03531-5

**Published:** 2024-06-25

**Authors:** Zhale Rajavi, Zahra Khorrame, Sadra Ashrafi

**Affiliations:** 1 Negah Aref Ophthalmic Research Center, Shahid Beheshti University of Medical Sciences, Tehran, Iran; 2 Ophthalmic Epidemiology Research Center, Research Institute for Ophthalmology and Vision Science, Shahid Beheshti University of Medical Sciences, Tehran, Iran; 3 Department of Ophthalmology, School of Medicine, Shahid Beheshti University of Medical Sciences, Tehran, Iran; 4 Ophthalmic Research Center, Research Institute for Ophthalmology and Vision Science, Shahid Beheshti University of Medical Sciences, Tehran, Iran

**Keywords:** Esotropia, Refractive status, Surgical outcome

## Abstract

**Background:**

The success of the strabismus surgery can hinge on several factors. One of these factors is refractive condition like hyperopia or myopia. Our study seeks to evaluate the surgical outcomes in patients with esotropia and myopia.

**Methods:**

This case-control study encompassed all surgical cases of esotropia at Torfe and Negah Hospital between 2016 and 2021, which satisfied our specified inclusion criteria. The initial variables from electronic medical records were collected, including demographic, clinical, and surgery-related factors. At the final follow-up appointment, the level of eye deviation, both at distance and near, was recorded. We considered the operation a “success” for patients with a post-surgery distance eye deviation of 10(Pd) or less. Patients with greater deviation were classified as surgery failure. Statistical analyses were executed using SPSS software (version 16.0), and a P-value less than 0.05 was considered significant.

**Results:**

Of the 194 patients evaluated, 112 were incorporated into the study. Surgical failure was observed in 14.29% of myopic patients, 29.79% of hyperopic patients, and 31.82% of emmetropic patients. The myopia group displayed a 0.19 odd ratio for surgical failure compared to the combined hyperopia and emmetropia groups, not statistically significant (OR: 0.19, CI 95%: 0.03–1.02). Additionally, patients diagnosed with Lateral Rectus Under-action were found to be 6.85 times more likely to experience surgery failure(OR: 6.85, CI 95%: 1.52–30.94). An elevated risk of surgical failure was also identified in patients who underwent Inferior Oblique Weakening procedure, indicated by a 3.77-fold increase in the odds ratio for failure(OR: 3.77, CI 95%: 1.08–13.17).

**Conclusion:**

In our study, despite numerical disparities, there was no statistical difference among the success rates of all esotropia patients with different refractive errors. The patients with LRUA or IOOA showed lower success rates. Myopic patients had higher post-op overcorrection with lower reoperation rates compared to hyperopic or emmetropic patients.

## Background

Strabismus, often referred to as “crossed eyes,” is a prevalent visual misalignment affecting both children and adults. This condition results in varied symptoms, such as diplopia, diminished vision, and decreased quality of life [1–3]. Surgical intervention is a primary therapeutic strategy for addressing strabismus, with a reported efficacy of approximately 80% [1, 4, 5]. However, the success of this surgery can hinge on several factors: the specific classification and intensity of strabismus, the age at which surgery is conducted, and associated refractive conditions like hyperopia or myopia [6, 7].

Hyperopia, a prevalent refractive error, impacts approximately 10% of the population [8]. This condition can prompt excessive convergence of the eyes, potentially leading to esotropia even post-strabismus surgery [9, 10]. Conversely, myopia, which affects around 30% of individuals [11], can induce eye divergence, culminating in exotropia following strabismus surgery [12]. The influence of these refractive errors on the outcomes of strabismus surgery remains a contentious topic within ophthalmology, with studies producing divergent findings on the matter. Research on the influence of hyperopia and myopia on strabismus surgery has yielded varied outcomes. Some studies suggest a lower success rate in hyperopic patients undergoing exotropia surgeries [6, 13], while others found diminished success in myopic individuals [14]. Furthermore, another study did not identify a noteworthy correlation between refractive errors and surgical outcomes in these patients [15]. Notably, a limited body of research addresses the effects of refractive errors on surgical outcomes in esotropic patients. A study conducted in 1997 suggests that the high myopic patients may require more extensive muscle resection or recession procedures to correct eye deviation effectively [16]. Another investigation into the association between hyperopia and the effectiveness of surgery for esotropia found no significant relationship between them [7].

The discrepancies observed in earlier studies and the limited research on how different types of refractive errors impact the outcomes of strabismus surgery in patients with esotropia emphasize the imperative for deeper investigation into the impact of refractive errors on strabismus surgery outcomes. Considering the widespread nature of these errors in the general population and their potential influence on surgical results, a more comprehensive understanding is essential. Our study seeks to evaluate the surgical outcomes in patients with esotropia by comparing those with myopia against a control group comprised of individuals with hyperopia or emmetropia.

## Methods

### Study setting and population

The present study employed a case-control design to investigate surgical cases of esotropia at Torfe and Negah Hospital from 2016 to 2021 that met our specified inclusion criteria. Patients with myopia were studied as cases, while those with hyperopia or emmetropia were treated as controls. Ethical approval for the study was granted by the Research Ethics Committees of Ophthalmic Research Center, Shahid Beheshti University of Medical Sciences, Tehran, Iran (ethical code: IR.SBMU.ORC.REC.1402.007).

**Fig. 1:**
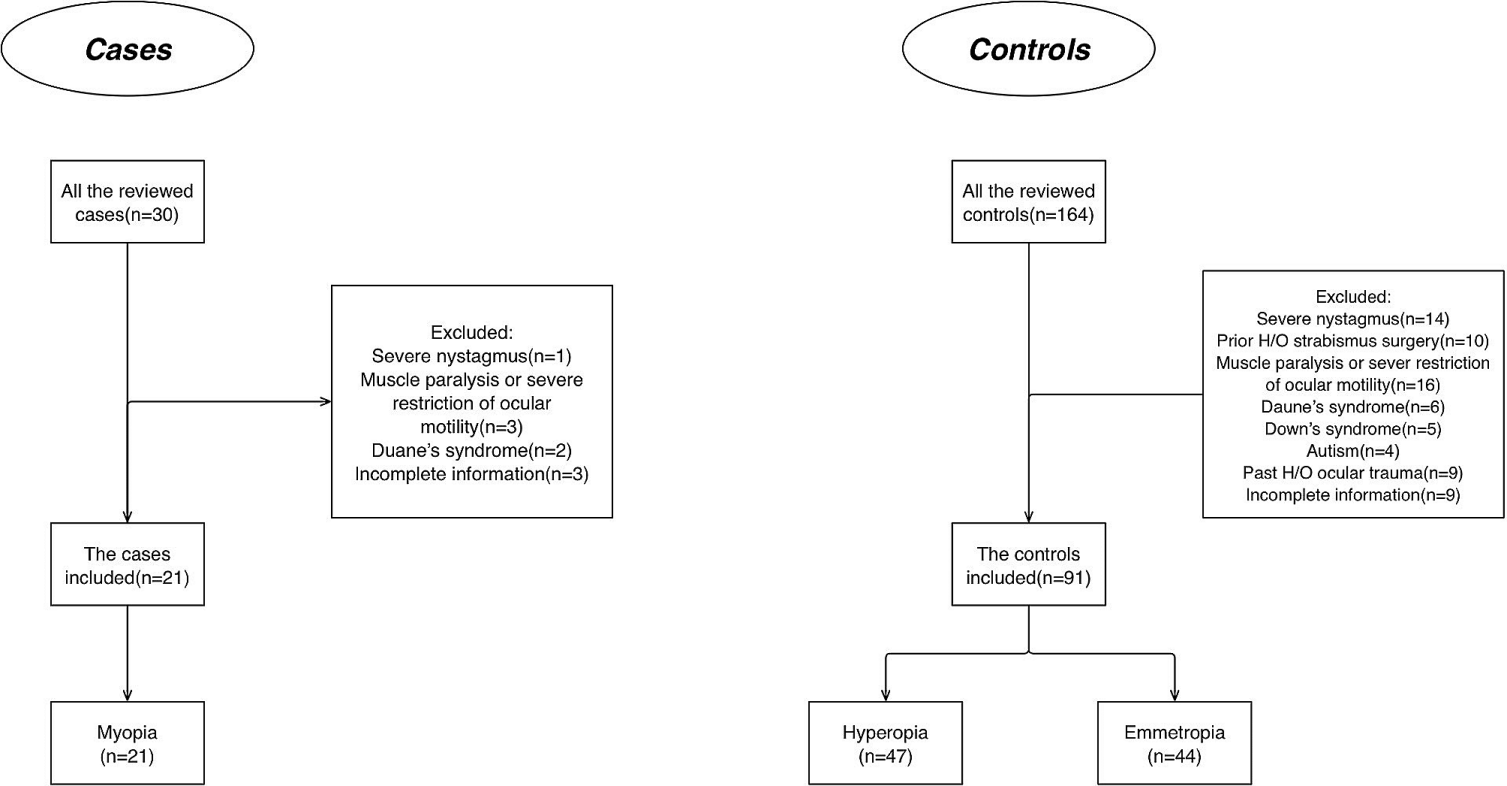
Flow diagram of the patients included in the study

In the current case-control study, we defined the case group as myopic patients diagnosed with acquired non-accommodative esotropia. These patients had undergone strabismus surgeries, including Bilateral Medial Rectus Recession (BMRREC), Unilateral Medial Rectus Recession (UMRREC), or Recession and Resection (R&R). Patients with esotropia and a high Accommodative Convergence/Accommodation (AC/A) ratio underwent surgery using the slant method, which involved recession of the medial rectus muscle at the superior pole for far esotropia and at the inferior pole for near esotropia. Myopia in these subjects was determined by a Sphere measurement of less than − 0.5 diopters in the eye exhibiting the highest refractive error (Fig. 1).

The control group comprised two subsets of patients: those with hyperopia and accommodative esotropia, and those with emmetropia and acquired non-accommodative esotropia. Both subsets in the control group had undergone strabismus surgeries similar to the case group. Hyperopia was characterized by a Sphere measurement exceeding + 2 diopters, while emmetropia was identified in patients with a Sphere measurement ranging between − 0.5 and + 2 diopters (Fig. 1). All surgeries were performed by Dr. Z.R, and all measurements were taken by a skilled optometrist who was blinded to the type of operation performed on the patients.

### Data collection

The patient data was sourced from electronic medical records. Initial variables collected included demographic factors like age, gender, and family history related to both eyeglasses usage and ocular deviation. Clinical metrics were also obtained, encompassing preoperative measures of both distant and near ocular deviation, muscle dysfunction indicators like lateral rectus underaction and inferior oblique overaction, and the type of deviation pattern—categorized as V pattern, A pattern, or no pattern. Additionally, the study evaluated AC/A ratio and the Best-Corrected Visual Acuity (BCVA) (measured in LogMAR units) and amblyopia.

The extent of ocular deviation was assessed using either the alternative prism cover test or the Krimsky test. A high AC/A was designated for those patients whose near deviation exceeded their far deviation by more than ten prism diopter (pd) [17]. Vision acuity was evaluated using the Snellen chart, and these results were then translated into LogMAR format for easier comparison. Based on prior research indicating its influence on postoperative deviation outcomes, vision from the weaker eye was utilized for study purposes [18]. Criteria for identifying amblyopia included either a minimum vision of less than 20/30 in at least one eye or a vision disparity greater than two lines between the two eyes [19].

Cyclopentolate 1% and Tropicamide 1% drops were administered to the subject’s eye to assess the refractive error. Approximately 30–45 min post-instillation, the refractive error was measured using an auto-refractometer. If the patient did not cooperate, retinoscopy was done. Based on the Sphere measurement of the eye with the higher refractive error, patients were classified into three categories: those with a Sphere greater than + 2 were considered hyperopic, those with a Sphere less than − 0.5 were labeled myopic, and all remaining patients were categorized as emmetropic. Additionally, if the difference in the spherical equivalent between the two eyes was more than one diopter, the condition was identified as Anisometropia.

We also collected details about each patient’s surgery from their medical records. This included the kind of surgery performed, such as UMRREC, BMRREC, or R&R, the total number of muscles operated on, whether slant surgery was performed for patients with high AC/A ratio, and if the surgery was accompanied by procedures like inferior oblique weakening(IOW) or muscle transposition.

### Outcome measurement

The surgical outcomes in our study were assessed based on the latest patient data available during follow-up, which was recorded at a minimum of six months post-operation. At this point, we recorded information like the level of eye deviation both at a distance and near and any complications after surgery, such as overcorrection, under-correction, or the need for additional surgery. Moreover, the early post-op deviation maximum in one week after surgery was also measured. We considered the operation a “success” for patients with a late post-surgery distance eye deviation of 10(Pd) or less. Patients with greater deviation were classified as surgery failure.

### Statistical analysis

Qualitative variables were characterized by frequency counts and percentages, while quantitative variables were summarized using means and standard deviations. (Mean ± SD). For comparing quantitative data between the two study groups, independent sample t-tests were employed. Chi-square tests were used to analyze differences in qualitative variables between these groups. To assess intra-group changes in quantitative variables pre- and post-surgery, paired sample t-tests were applied. For intra-group comparisons of qualitative variables, McNemar’s tests were conducted. Factors contributing to surgical failure were ascertained via multivariable logistic regression, from which odds ratios and 95% confidence intervals were calculated. Statistical analyses were executed using SPSS software (version 16.0), and graphical representations were generated using Prism software (version 8.0.1). Statistical significance was considered for p-values less than 0.05.

## Results

This research focused on individuals who underwent surgery for esotropia between 2016 and 2021. Out of a cohort of 194 patients, several were omitted from the analysis for specific reasons. The details are shown in the study chart (Fig. 1).

Table 1 shows that upon pairwise comparison, no statistically significant differences were observed in age or gender among the groups (Myopia vs. hyperopia; Myopia vs. emetropia) (*P* > 0.05). In contrast, certain other variables showed marked differences between these groups. Specifically, a family history of eye deviation was present in 27.7% of hyperopic (*P* = 0.006) and 20.4% of emmetropic patients (*P* = 0.02) but absent in the myopic group. Additionally, the anisometropic patient proportion was higher in the myopic group, with 7(33.3%) individuals, compared to 3(6.8%) in the emmetropic group(*P* = 0.01). Furthermore, the rate of patients with LRUA was considerably greater in the myopic group at 42.85%, compared to 18.8% in the emmetropic group(*P* = 0.03) (Table 1).

**Table 1:** Demographic and clinical characteristics of myopia, hyperopia and emetropia groups

Variables			Case	Controls					Total(*n* = 112)
			Myopia(*n* = 21)	Hyperopia(*n* = 47)		*P*-value^†^	Emetropia(*n* = 44)	*P*-value^††^	
Sex	Male		8(38.09%)		28(59.57%)	0.08	21(47.72%)	0.46	57(50.90%)
	Female		13(61.91%)		19(40.43%)		23(52.28%)		55(49.10%)
Age(year)			8.79 ± 6.48	6.03 ± 3.97		0.08	7.85 ± 7.17	0.61	7.26 ± 5.93
Family history of glasses(yes)			9(42.85%)	13(27.66%)		0.21	21(47.72%)	0.71	43(38.40%)
Family history of ST(yes)			0(0%)	13(27.66%)		0.006*	9(20.45%)	0.02*	22(19.64%)
H/O incubator care(yes)			0(0%)	5(11.90%)		0.31	7(15.91%)	0.08	12(10.71%)
Worse eye BCVA(LogMAR)			0.33 ± 0.36	0.27 ± 0.26		0.47	0.20 ± 0.25	0.16	0.25 ± 0.28
Amblyopia(at least one eye) (yes)			11(55%)	23(58.97%)		0.77	21(52.5%)	0.85	55(49.10%)
SE(higher eye)(D)			-4.36 ± 3.80	4.13 ± 1.84		< 0.001**	0.93 ± 0.72	< 0.001**	1.28 ± 3.70
Anisometropia(yes)			7(33.33%)	9(19.14%)		0.20	3(6.8%)	0.01*	19(16.96%)
Pre-op high AC/A(yes)			8(38.09%)	12(25.53%)		0.29	12(27.27%)	0.37	32(28.57%)
IOOA(yes)			13(61.90%)	29(61.70%)		0.98	20(45.45%)	0.21	62(55.35%)
LRUA(yes)			9(42.85%)	10(21.27%)		0.06	8(18.18%)	0.03*	27(24.10%)
Fundus examination(Abnormal)			5(23.81%)	8(17.02%)		0.51	5(11.36%)	0.19	18(16.07%)
Pattern		V-P	7(33.33%)	25(53.19%)		0.09	20(45.46%)	0.31	52(46.42%)
		A-P	0(0%)	3(6.38%)			1(2.27%)		4(3.57%)
		No-P	14(66.67%)	19(40.43%)			23(52.27%)		56(50.01%)

Frequency is reported for categorical variables and Mean ± SD for continues variables

ST, strabismus; P, pattern; BCVA, best corrected visual acuity; AC/A, accommodative convergence /accommodation (AC/A); IOOA, inferior oblique over action; LRUA, lateral rectus under action

*Chi-square test for categorical variables

**Independent T-test for continues variables

† Myopia Vs. Hyperopia

††Myopia Vs. Emetropia

Table 2 presents a comparison of surgical variables among the various groups. The data indicates that no statistically significant differences were observed between the groups in different crucial variables (Table 2).

**Table 2:** Surgery-related characteristics of myopia, hyperopia and emetropia groups

Variables		Case	Controls				Total(*n* = 112)
		Myopia(*n* = 21)	Hyperopia(*n* = 47)	*P*-value^†^	Emetropia(*n* = 44)	*P*-value^††^	
Pre-op far deviation(Pd)		31 ± 17.66	32.14 ± 13.30	0.768	30.61 ± 9.73	0.926	31.33 ± 12.91
Pre-op near deviation(Pd)		37.42 ± 13.35	38.89 ± 13.91	0.686	34.79 ± 9.53	0.366	37.00 ± 12.29
Type of Surgery	UMRREC	16(76.19%)	41(87.23%)	0.157	34(77.27%)	0.270	91(81.3%)
	BMRREC	1(4.76%)	3(6.38%)		9(20.45%)		13(11.6%)
	R&R	4(19.04%)	3(6.38%)		1(2.27%)		8(7.1%)
Number of muscles		2.24 ± 0.70	2.66 ± 1.09	0.061	2.41 ± 0.89	0.446	2.48 ± 0.96
IOW(Yes)		6(28.57%)	16(34.04%)	0.658	8(18.18%)	0.344	30(26.8%)
Slant(Yes)		7(33.33%)	7(14.89%)	0.0.85	6(13.63%)	0.065	20(17.9%)
Transposition(Yes)		3(14.28%)	4(8.51%)	0.472	5(11.36%)	0.706	12(10.7%)
Follow-up duration(months)		42 ± 57.15	19.42 ± 16.8	0.089	18.01 ± 16.03	0.073	23.10 ± 29.64

Frequency is reported for categorical variables and Mean ± SD for continues variables

UMRREC, unimedial rectus recession; BMRREC, bimedial rectus recession; R&R, recession and resection; IOW, inferior oblique weakening

*Chi-square test for categorical variables

**Independent T-test for continues variables

† Myopia Vs. Hyperopia

††Myopia Vs. Emetropia

In Table 3, we present the results of the extent of the late and early far deviation and the occurrence of a high AC/A ratio in patients both pre- and post-surgery. In all categories, a significant decrease in the far deviation was observed after the surgical procedure compared to the preoperative assessments(*P* < 0.001). In the same way, the results indicated a decrease in the population of patients displaying a high AC/A ratio across all experimental groups. The observed decrease in patients with myopia(*P* = 0.03) and emmetropia(*P* = 0.03) demonstrated statistical significance, whereas the decrease in patients with hyperopia(*P* = 0.549) did not achieve statistical significance (Table 3).

**Table 3:** Pre and post-op far and near deviation, and high AC/A in myopia, hyperopia and emetropia groups

Groups/Variables	Case	Controls				Total(*n* = 112)	*P*-value
	Myopia(*n* = 21)	Hyperopia(*n* = 47)	*P*-value††	Emetropia(*n* = 14)	*P*-value†††		
Pre-op far deviation	32.25 ± 17.14	32.15 ± 13.30	0.768**	30.61 ± 9.73	0.926**	31.56 ± 12.74	0.847**
Early post-op far deviation	1.86 ± 2.87	2.91 ± 2.69	0.147**	2.09 ± 3.23	0.779**	2.39 ± 2.96	0.273**
Late post-op far deviation	6.8 ± 13.78	7.53 ± 8.50	0.792**	8.35 ± 9.82	0.612**	7.72 ± 10.06	0.842**
*P*-value^†^	< 0.001*	< 0.001*	-	< 0.001*	-	< 0.001*	-
Pre-op high AC/A(Yes)	8(38.09%)	12(25.53%)	0.294^##^	12(27.27%)	0.377^##^	32(28.57%)	0.554^##^
Post-op high AC/A(Yes)	2(9.52%)	9(19.15%)	0.319^##^	4(9.09%)	0.955^##^	15(13.39%)	0.314^##^
*P*-value^†^	0.031^#^	0.549^#^	-	0.039^#^	-	0.003^#^	-

**Frequency** is reported for categorical variables and **Mean ± SD** for continues variables

AC/A, accommodative convergence/accommodation.

*Paired-Samples T test

**Independent Sample T test

^#^ McNemar test

^##^Chi-square test

† Pre Vs. Post

† Myopia Vs. Hyperopia

††† Myopia Vs. Emetropia

**Fig. 2:**
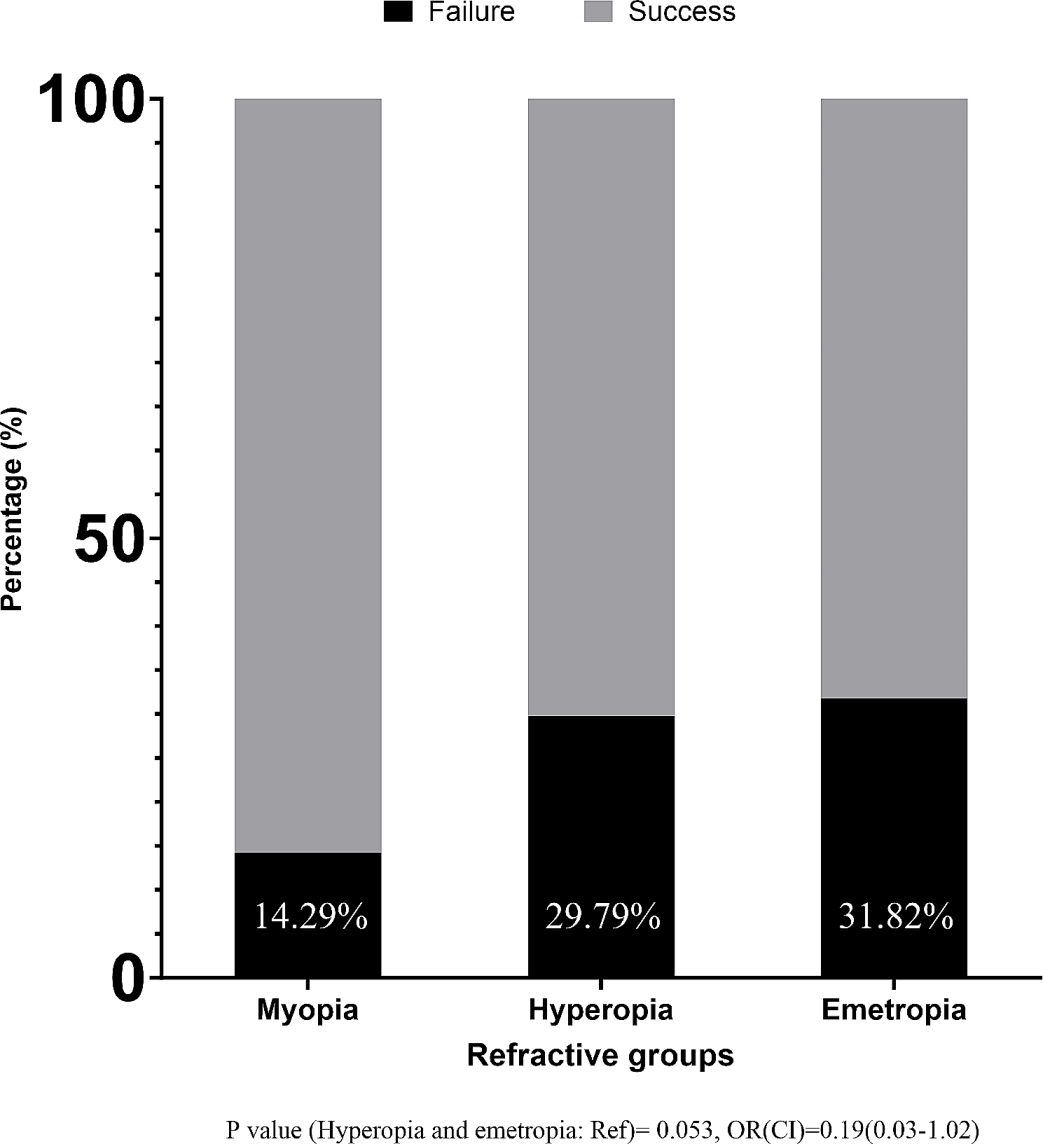
Success and failure rates according to the refractive status

**Fig. 3:**
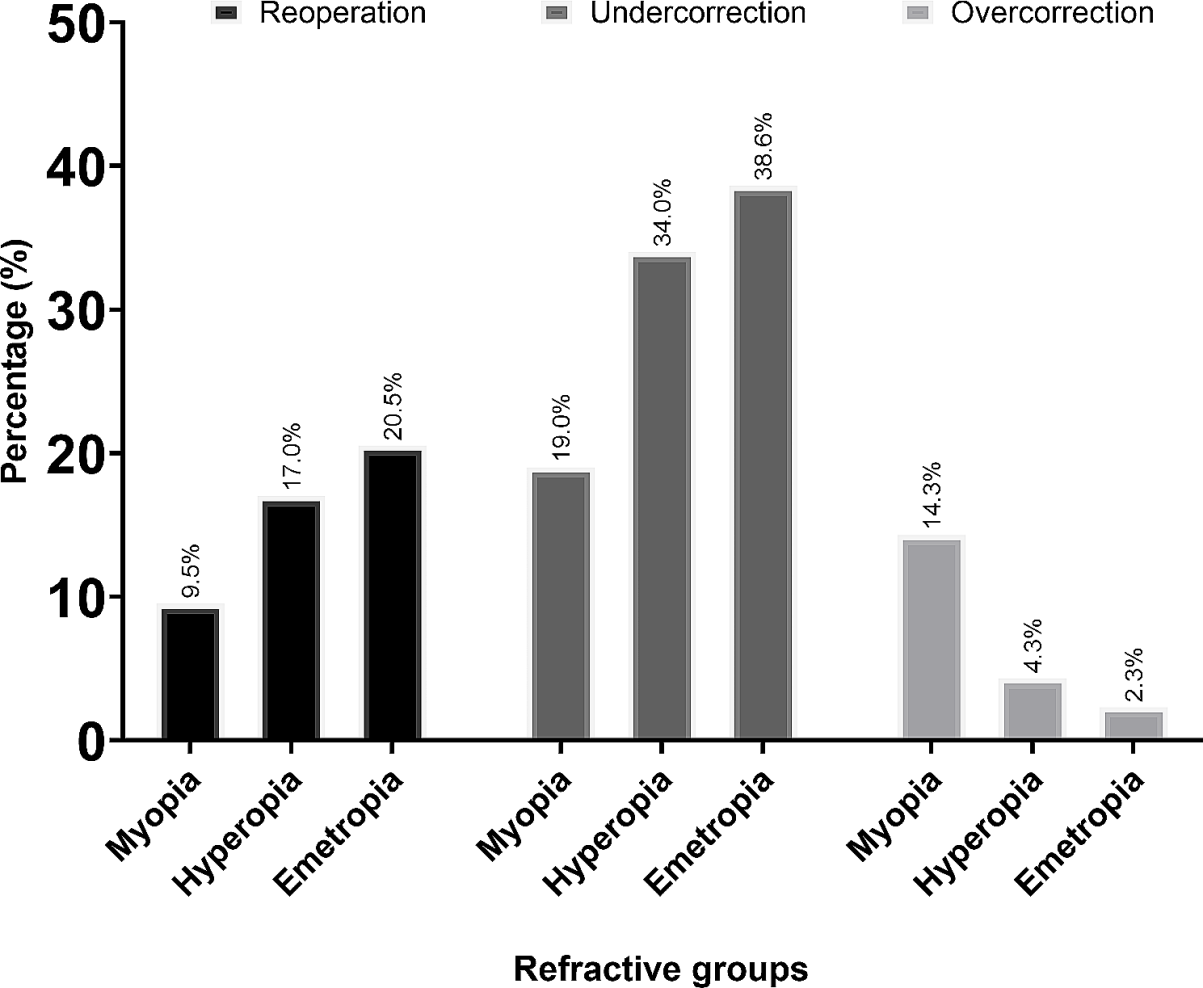
Under and over correction, and reoperation according to refractive status

In the study’s outcomes depicted in Fig. 2, we observed differences in surgical success and failure among the myopic, hyperopic, and emmetropic groups. The myopic patients demonstrated a lower failure rate of 14.3%, compared to 29.8% and 31.8% for the hyperopic and emmetropic groups, respectively. Despite these numerical disparities, statistical analysis revealed no significant differences between the groups (*P* > 0.05) (Fig. 2). Figure 3 delves into postoperative complications, subdivided into overcorrection, under-correction, and the necessity for a reoperation. Myopic patients were more likely to experience overcorrection, with a rate of 14.3%, compared to a mere 4.3% and 2.3% in the hyperopic and emmetropic subjects. However, the rate of under-correction was lower in the myopic group, with a rate of 19.0%, as opposed to 38.6% and 34.0% in their hyperopic and emmetropic patients. Additionally, the requirement for subsequent surgery was least in myopic patients at 9.5%, while 17% of hyperopic and 25% of emmetropic individuals needed the reoperation. Again, these differences did not reach statistical significance (*P* > 0.05) (Fig. 3).

In the multivariable logistic regression analysis results, we observed several noteworthy associations with surgical failure. The myopia group displayed a 0.19 odds ratio for surgical failure compared to the combined hyperopia and emmetropia groups, not statistically significant (OR: 0.19, P: 0.053). Additionally, patients diagnosed with LRUA were found to be 6.85 times more likely to experience surgery failure(OR: 6.85, P: 0.012). A heightened risk of surgical failure was also identified in patients who underwent IOW procedures, indicated by a 3.77-fold increase in the odds ratio for failure(OR: 3.77, P: 0.038). Though not reaching statistical significance, other findings included a 0.79 elevation in the likelihood of surgical failure in patients undergoing R&R surgery(OR: 1.79, P: 0.586), as well as a 0.37 increase among those with amblyopia(OR: 1.37, P: 0.557) (Table 4).

**Table 4:** Multivariable logistic regression models of factors associated with surgery failure in esotropia patients undergoing surgery

Variables		Failure(*n* = 31)	OR(CI)	*P*-value
**Demographic**				
Age		6.03 ± 5.17	0.99(0.89–1.10)	0.877
**Clinical**				
Refractive group	Myopia(*n* = 21)	3(14.29%)	0.19(0.03–1.02)	0.053
	Emetropia/Hyperopia(*n* = 91)	28(30.77%)	Ref	-
Amblyopia(Ref: no)		16(57.14%)	1.37(0.48–3.88)	0.557
Anisometropia(Ref: no)		3(9.7%)	0.20(0.04–1.07)	0.060
Pre-op high AC/A(Ref: no)		9(29.03%)	2.38(0.71–7.95)	0.160
LRUA(Ref: no)		12(38.71%)	6.85(1.52–30.94)	0.012
**Surgery-related**				
Pre-op Far deviation		30.90 ± 11.38	1.02(0.97–1.06)	0.447
Type of surgery	R&R(*n* = 8)	3(37.50%)	1.79(0.22–14.45)	0.586
	BMRREC(*n* = 13)	3(23.08%)	1.08(0.19–5.96)	0.931
	UMRREC (*n* = 91)	25(27.47%)	Ref	-
IOW(Ref: no)		10(32.26%)	3.77(1.08–13.17)	0.038

OR, odd ratio; CI, confidence interval; AC/A, accommodative convergence /accommodation; LRUA, lateral rectus under action; UMRREC, unimedial rectus recession; BMRREC, bimedial rectus recession; R&R, recession and resection; IOW, inferior oblique weakening

## Discussion

Our findings indicate that myopic patients with esotropia had statistically similar success rate in comparison to those who are hyperopic (Sphere > + 2) or emmetropic (-0.5 ≤ Sphere ≤ + 2) with esotropia. While overcorrection appeared more commonly in the myopic group, undercorrection was less prevalent in them, compared to the control group. However, these variations between groups were not statistically significant. It is noteworthy to highlight that LRUA and IOW were identified as major risk factors contributing to surgical failure in these groups.

In the current study, it was observed that myopic patients exhibited a reduced rate of operation failure, with these patients having an 81% decreased likelihood of such failures. However, this finding was not statistically significant. A review of prior research revealed limited exploration of the impact of refractive status on surgical outcomes in esotropic patients. Notably, Shauly et al. delved into this topic, suggesting that high myopia patients with infantile esotropia might require more extensive muscle recession or resection compared to their hyperopic and emmetropic counterparts to achieve equivalent postoperative outcomes [16]. However, their study primarily focused on the alteration in deviation after the surgery with muscle recession amounts, without evaluating long-term success or failure rates. A possible rationale behind these findings might be the increased axial length in high myopic individuals. This might provide them with enhanced muscle leverage, resulting in diminished post-operative muscle retraction forces [20]. Moreover, due to the elongated muscles characteristic of myopic patients, more significant muscle recess might be mandated to match the eye displacement seen in hyperopic and emmetropic patients. Given the scarcity of investigations on esotropic patients in this context, further research is warranted. While research focusing on esotropic patients remains limited, a more substantial number of studies have been undertaken regarding exotropic patients with hyperopia [6, 13–15, 21].

In this study, we noted a heightened frequency of overcorrection among myopic patients in comparison to their hyperopic and emmetropic counterparts. This trend can be attributed to the application of a negative lens for myopic individuals, which amplifies the apparent deviation of the eye [15]. As a result, the likelihood of post-operative overcorrection becomes more pronounced in this group.

In our analysis, among the various factors examined, both LRUA and inferior oblique overaction were identified as significant contributors to surgical failure. This aligns with findings from Rajavi et al., where LRUA was highlighted as a risk determinant for unsuccessful surgeries and subsequent reoperations for esotropia [22]. Similarly, Scelfo et al. found that combining inferior oblique myectomy with lateral rectus recession in exotropic patients heightened the need for movement readjustment due to overcorrection [23]. This challenge arises as operating on multiple muscles complicates the precise determination of the extent of each muscle’s recession or resection required to achieve accurate eye position, consequently elevating the risk of surgery failing. Corroborating this, our study noted that R & R surgeries, encompassing two muscles, posed a higher surgical risk than UMRREC, which only involves one muscle, though this observation was not statistically significant.

These findings underline the complex relationship between refractive errors and specific ocular motor anomalies in determining surgical outcomes, emphasizing the need for comprehensive preoperative assessments that go beyond refractive error to include detailed ocular motility examinations. Additionally, our data hint at the potential advantage of slightly overcorrecting esotropic patients with hyperopia and emetropia, or with risk factors like LRUA and IOOA. While promising, this approach should be considered with caution, balancing the benefits against the risks of possible ocular complications, and should be tailored to each patient’s unique condition. Our findings suggest a direction for future research to further understand and optimize treatment strategies for esotropia, contingent upon a deeper exploration and professional consensus on these approaches.

Notably, patient age at the time of surgical intervention was not observed to play a significant role in determining the surgery’s success or failure. This is consistent with findings from Ahn et al., who similarly reported that the patient’s age at the time of surgery did not impact the surgical outcome [13]. Several other prior studies have corroborated this observation [14, 24, 25]. On a different note, the extent of preoperative deviation was also found to be non-influential in determining surgical outcomes. This mirrors the findings of Ahn et al., who noted analogous results in exotropic patients [13]. However, it’s worth noting the discrepancy in the literature, as several other studies have emphasized preoperative deviation as a crucial prognostic factor for surgery [6, 26, 27]. Given these varying conclusions, it becomes imperative to weigh the role of deviation in pre-surgical evaluations. More extensive research is warranted to elucidate its true impact on surgical outcomes in strabismus surgery. Interestingly, variables like amblyopia, anisometropia, and pre-op high AC/A did not emerge as influential determinants for surgical failure in our study.

A primary limitation of this study was the limited sample size of operated esotropic patients with myopia, due to their rareness. Additionally, there were disparities in a few baseline attributes among the various groups, which may affect our outcomes. The inconsistency in surgical success metrics across various studies combined with the scant research on esotropic patients complicates direct comparisons with prior literature. It would be prudent for future studies to place a greater emphasis on esotropic patients to bridge this research gap.

## Conclusion

In our investigation, surgeries conducted on esotropic patients with myopia exhibited a statisticaly similar success rate compared to those with hyperopia and emmetropia. Notably, lateral rectus under action and inferior oblique over action emerged as the two predominant risk factors for surgical failure. These findings not only provide valuable insights into the surgical outcomes across different refractive error groups but also emphasize the need for clinicians to be vigilant of these specific preoperative findings. Recognizing and addressing these risk factors can enhance surgical planning and potentially improve the success rate, thus benefiting patient outcomes in clinical settings.

## Data Availability

Data are available from the corresponding author upon a reasonable request.

## Abbreviations

BMRREC: Bilateral Medial Rectus Recession
UMRREC: Unilateral Medial Rectus Recession
R&R: Recession and Resection
BCVA: Best-Corrected Visual Acuity
AC/A: Accommodative Convergence/Accommodation
IOW: Inferior Oblique Weakening
LRUA: Lateral Rectus under-action
Op: Operation
SD: Standard deviation
OR: Odd ratio
